# Rising epidemic of HIV-1 infections among general populations in Fujian, China

**DOI:** 10.1186/1742-4690-9-S2-P138

**Published:** 2012-09-13

**Authors:** H Wang, X Yao, P Yan, Z Chen

**Affiliations:** 1 AIDS Institute, LKS Faculty of Medicine, The University of Hong Kong, Hong Kong, Hong Kong; 2 Fuzhou Center for Disease Control and Prevention, Fuzhou, China; 3 Fujian Center for Disease Control and Prevention, Fuzhou, China

## Background

Monitoring HIV transmission and viral diversity has significant impacts on guiding effective vaccine development. Until now, few large-scale studies have investigated HIV infections among the general populations of China.

## Methods

915,830 and 2,152,658 blood samples from various groups were collected in 2006-2007 and 2008-2009, respectively, in Fujian, a low prevalent region in China. Comprehensive HIV-1 epidemiology and molecular epidemiology studies were conducted.

## Results

Our data revealed a significant rise of the overall prevalence of infections within a short time period, from 0.064% in 2006-2007 to 0.074% in 2008-2009 (p=0.003), which resulted in the double numbers of infections from 528 in 2006-2007 to 1129 in 2008-2009. Critically, the prevalence rate among general populations such as voluntary blood donors, recipients of blood transfusion and people during pre-surgery screening had significantly increased in recent years (p<0.001). Besides CRF01_AE as the dominant circulating subtype (61/86, 70.9%), 25 non-CRF01_AE strains were found contributing to increased HIV-1 genetic diversity including C/CRF07_BC/CRF08_BC (5.8%), B/B’ (15.1%) and unique recombinant forms (8.1%). More than 30% (26/81) of subjects were found to contain various drug-resistant mutations.

## Conclusion

The rising epidemic in recent years in Fujian is likely due to the increased prevalence of HIV-1 infections among general populations and multiple viral subtypes circulating. Our findings will be useful for helping to enhance the current surveillance system and to generate strategic prevention programs targeting general populations in China. Moreover, these results also have implications for AIDS vaccine research.

